# Analysis of the Potential Mediating Role of Plasma Proteins in the Association Between Air Pollution Exposure and Lung Cancer Risk Among Ever-Smokers

**DOI:** 10.1021/acs.est.6c07407

**Published:** 2026-10-19

**Authors:** Ziyin Tang, Donghai Liang, Jeremy A. Sarnat, W. Ryan Diver, Todd M. Everson, Emily Deubler, Youran Tan, Aparna H. Kesarwala, Michelle C. Turner, Mattias Johansson, Hilary A. Robbins, Carmen J. Marsit, Alpa V. Patel, Ying Wang

**Affiliations:** † Gangarosa Department of Environmental Health, 1371 Rollins School of Public Health, Emory University, Atlanta, Georgia, United States 30322; ‡ Department of Population Science, American Cancer Society, Atlanta, Georgia, United States 30303; § Barcelona Institute for Global Health (ISGlobal), Barcelona, Spain 08003; ∥ Universitat Pompeu Fabra (UPF), Barcelona, Spain 08003; ⊥ Department of Radiation Oncology, Emory University School of Medicine, Atlanta, Georgia 30322, United States; # CIBER Epidemiología y Salud Pública (CIBERESP), Madrid, Spain 28029; ¶ 56140 Genomic Epidemiology Branch, International Agency for Research on Cancer, Lyon, France 69366

**Keywords:** air pollution, mixture, lung cancer, proteins, inflammation, immunity

## Abstract

The biological mechanisms linking air pollution to lung carcinogenesis remain underexplored. This study aimed to identify key proteins and pathways involved in mediating the effects of air pollutants on lung cancer risk. We analyzed 95 matched lung cancer case–control pairs of ever-smokers with prediagnosis plasma protein profiles measured in the Cancer Prevention Study-II Nutrition Cohort. We assessed associations between protein levels and six individual air pollutants, air pollution mixture, and lung cancer risk, adjusting for confounders and multiple testing. We identified overlapping proteins and enriched pathways associated with both exposures and lung cancer risk, followed by an exploratory high-dimensional mediation analysis (HIMA) to investigate the potential mediating roles of proteins. Seventeen proteins were associated with both exposures and lung cancer, including CX3CL1, ADAM15, and CXCL17 (*P* < 0.05). HIMA identified six proteins, including CX3CL1 and CXCL17, as potential mediating proteins of individual pollutant-lung cancer associations (Bonferroni-corrected *P* < 0.05), while these findings did not remain significant when excluding cases diagnosed within one year of blood draw. The exploratory results suggest that proteins and pathways related to inflammatory chemokine signaling, immune modulation, and altered cell proliferation, migration, and survival may play significant roles in air pollution-lung cancer association among ever-smokers.

## Graphical abstract

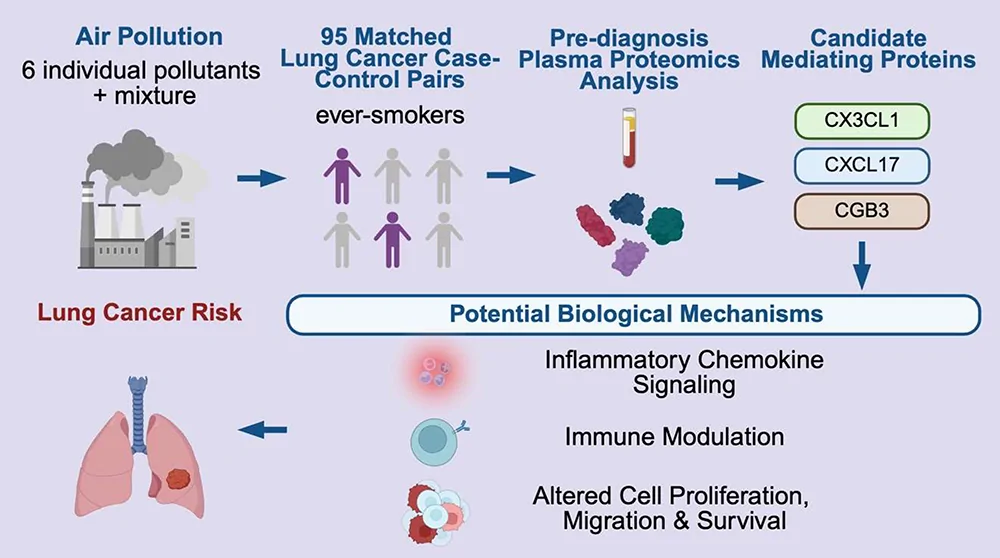

## Introduction

Outdoor air pollution is an established and critical environmental risk factor for lung cancer. Numerous studies, including those conducted within the Cancer Prevention Study-II (CPS-II) cohort, have reported associations between exposure to ambient air pollutants and increased lung cancer incidence and mortality.^1–8^ In 2013, the International Agency for Research on Cancer classified ambient air pollution and particulate matter (PM) as Group 1 human carcinogens.^9^ Inhalable air pollutants deposit in the respiratory tract, causing direct damage. Additionally, they induce persistent low-grade oxidative stress and inflammation, both locally and systemically.^10^ Other potential processes include activation of oncogenes and inactivation of tumor suppressor genes,^11^ cell cycle alterations,^12–14^ induction of genomic instability,^15^ suppression of apoptosis,^10^ and dysregulation of energy metabolism.^16^ However, detailed molecular mechanisms and biological pathways driving these processes have not been thoroughly investigated, due in part to the lack of sensitive biomarkers.

High-throughput omics approaches have shown great potential in uncovering molecular mechanisms underlying environmental exposures and cancer etiology.^17^ Previously, our group investigated metabolic pathways associated with air pollution exposure and lung cancer risk separately within the CPS-II and CPS-3 cohorts using metabolomics—a comprehensive profiling of small molecules in biological samples.^18,19^ We found various air pollutants associated with plasma metabolites involved in lipid and amino acid metabolism, implicating pathways related to oxidative stress, systemic inflammation, energy metabolism, and signal transduction.^19^ Additionally, we found that similar metabolic pathways including lipid, amino acid, and xenobiotic metabolism were associated with lung cancer risk, with patterns varying by smoking status.^18^ However, metabolomics characterizes only a snapshot of cellular activities. Proteomics, the comprehensive study of proteins (i.e., the functional molecules of nearly all biological processes in the human body^20^) provides complementary insights. One focus of proteomics is quantifying protein abundance, which is assumed linked to cellular function.^21^ This approach complements metabolomics by identifying proteins and biological processes for changes in metabolite levels. Recently, we performed a proteome-wide analysis of air pollution exposure and identified proteins and pathways involved in immune responses, inflammation, cell signaling, and cell growth.^22^ Notably, several protein–pollutant associations differed by smoking history. However, it remains unclear whether these air pollution-related proteins and pathways play a role in lung carcinogenesis.

Previous studies have separately linked air pollution exposures to circulating proteomic profiles and identified circulating proteins associated with subsequent lung cancer risk. To our best knowledge, only one study has utilized proteomics to explore the mechanistic links between air pollution exposures and lung cancer risk.^23^ However, the prior study was based on tumor tissue collected from patients with established disease. In contrast, the present study examined prediagnostic plasma proteomic profiles from participants who were cancer-free at blood collection, providing an opportunity to investigate molecular alterations associated with air pollution exposure and future lung cancer risk before clinical diagnosis. Additionally, the previous study did not consider the joint effects of multiple air pollutants as a mixture or systematically examined the potential mediating role of proteins in this association. To address these gaps, we conducted a comprehensive investigation into the associations between six ambient air pollutants—assessing both individual and joint effects, plasma protein profiles, and lung cancer risk. Additionally, we explored the potential mediating role of plasma proteins in the relationship between air pollution exposure and lung cancer risk among current and former smokers in the well-characterized CPS-II Nutrition Cohort. Our goal was to characterize proteins and biological pathways that may lie along hypothesized pathways linking ambient air pollution exposure to lung cancer risk.

## Methods

### Study Design and Population

The study participants were drawn from the CPS-II Nutrition Cohort, a prospective cohort of 184,194 participants with a median age of 63 years from 21 U.S. states. Participants provided informed consent and completed a mailed baseline questionnaire to provide comprehensive demographic, medical, and behavioral information in 1992–1993. Follow-up surveys were sent to participants in 1997 and every other year thereafter. Between 1998 and 2001, about 40,000 participants provided a nonfasting blood sample at community hospitals. The blood samples were shipped overnight with coolant packs to a central repository, where they were aliquoted and stored at −130 °C for long-term preservation and future analysis. Lung cancer cases were first identified via self-report and were then validated using medical records, state cancer registry data, or National Death Index linkage (defined by ICD-10 codes C33 and C34, excluding histology codes ≥ 9590). This study protocol was reviewed and approved by the Emory University Institutional Review Board (Atlanta, GA) under approval number IRB00045780. The details of the cohort were published elsewhere.^24^

We initially included 115 matched lung cancer case–control pairs from a prior study investigating plasma protein markers of imminent lung cancer in individuals with a smoking history.^25,26^ All lung cancer cases were diagnosed within three years of blood draw. Controls were matched to cases on age at blood draw, date of blood draw, gender, and race. We excluded participants due to (1) protein profile outliers (N = 3); (2) missing air pollution data due to missing residential history information (N = 9); (3) missing covariate information (N = 11); and (4) matched case/control exclusions related to the above criteria (N = 18) (Figure S1). The final sample included a total of 190 participants (i.e., 95 matched lung cancer case–control pairs).

The goal of this study was to assess plasma proteins as potential mediating proteins in the association between air pollution and lung cancer risk. The analysis involved two components: assessing the relationship between air pollution and proteins, and the relationship between proteins and lung cancer risk. Both associations have been thoroughly investigated in prior work,^22,26^ including analyses that included data from five additional cohorts within the Lung Cancer Cohort Consortium. For the current study, we repeated these analyses in the current study population to enable their integration into a mediation framework.

### Retrospective Air Pollution Exposure Measurement

A detailed description of the retrospective air pollution exposure assessment has been provided in our previous analysis of the association between air pollution exposures and plasma proteins.^22^ Briefly, air pollution exposure levels were retrieved from the Center for Air, Climate, and Energy Solutions (CACES) database and linked to each participant based on the residential address at the census block level. The residential address was collected at the time of blood draw. The CACES database provided annual average exposure levels of six criteria air pollutants, including fine particulate matter with 2.5 μm or less in diameter (PM_2.5_), particulate matter with 10 μm or less in diameter (PM_10_), nitrogen dioxide (NO_2_), daily 8 h maximum ozone (O_3_), sulfur dioxide (SO_2_); carbon monoxide (CO), across the contiguous U.S. using integrated empirical geographic regression models.^27^ Since all pollutant data became available in 1999 and showed high correlations across 1999 to 2001 with correlation coefficients ranging from 0.84 to 0.97,^22^ we used exposure measurement in 1999 as proxies for 1998 (N = 6) to ensure consistency across all pollutants.

### Plasma Proteomic Profiling

Proteomics profiling was conducted in plasma samples using the Olink proteomics platform.^28^ Relative concentrations of 552 proteins were measured on six panels: Cardiovascular III, Inflammation, Immuno-oncology, Oncology II, Oncology III, and Neuro Exploratory. Cases and controls were randomly assigned to plates, with matched pairs placed on the same plate whenever possible. Internal controls were included in each sample to assess assay performance and sample quality, and samples failing quality control were excluded. Additional details on plasma proteomic profiling have been described elsewhere.^22,25^

The intra-assay coefficient of variance (CV) ranged from 3% to 7% (reference: <15%), and the interassay CV ranged from 7% to 11% (reference: <25%), indicating high data quality. For proteins measured on multiple panels, the measurement with the highest variance was retained. Proteins with more than 20% of values below the limit of detection (LOD) were excluded. However, no proteins met this criterion. For proteins with more than 80% detectable values, measurements below the LOD were imputed as LOD divided by the square root of 2. Relative protein levels were then log_2_-transformed and autoscaled to approximate a normal distribution. A total of 484 distinct proteins were included in the present analysis. Potential outliers were identified using the Ensemble Methods for Outlier Detection (EnsMOD) software.^29^ Three samples flagged as outliers were excluded from the analyses.

### Statistical Analysis

We conducted a descriptive analysis of covariates, summarizing continuous variables as arithmetic mean (standard deviation) and categorical variables as frequency (n) and percentage (%). Differences between cases and controls were assessed using the Wilcoxon rank sum test for continuous variables and Pearson’s Chi-squared test or Fisher’s exact test for categorical variables.

#### Association Between Air Pollution Exposures and Plasma Proteins

We employed two approaches to assess the associations between air pollution exposure and plasma proteins. First, we explored the overall effect of air pollution mixture on each plasma protein using quantile g-computation models. The standardized log_2_-transformed protein levels were regressed on the annual average level of all air pollutants, adjusting for age at blood draw (continuous), gender (categorical: male, female), body mass index (BMI) (categorical: <18.5 kg/m^2,^18.5 to <25 kg/m^2^, 25 to <30 kg/m^2^, ≥30 kg/m^2^), education level (categorical: less than high school, high school graduate, some college or associate’s degree, Bachelor’s degree and above), alcohol consumption (categorical: not current drinker, <1 drink/day, 1 drink/day, 2 or more drinks/day, unknown), fruit and vegetable consumption (categorical: first, second, third, and fourth quantile, unknown), smoking status (categorical: former, current smoker), and pack-years (continuous). Briefly, the covariate selection was informed by a comprehensive literature review and a directed acyclic graph, coupled with stepwise selection. The detailed selection process, definition, and collection time of covariates can be found elsewhere.^22^ By default, the effect estimates were expressed as changes in standardized log_2_-transformed proteins levels per one quartile (25%) increase in all air pollutant levels. Second, we assessed the effect of individual air pollutants on each plasma protein using multiple linear regression models among the total sample of combined cases and controls. The standardized log_2_-transformed protein levels were regressed on the annual average level of each air pollutant, adjusting for the same set of covariates above. To be comparable with mixture results, the effect estimates were expressed as changes in standardized log_2_-transformed protein levels per 1/2 interquartile range (IQR) increase in air pollutant levels. Significance for the air pollution−protein association analyses was assessed using nominal P < 0.05, with Benjamini-Hochberg (BH)-corrected false discovery rate (FDR) <0.2 reported in parallel; the two thresholds are reported alongside one another and were not jointly required.

#### Association Between Plasma Proteins and Lung Cancer Risk

We used conditional logistic regression to investigate the association between the level of each plasma protein and lung cancer risk. The cancer status was regressed on the standardized log_2_-transformed concentration of each protein, conditioning on matching variables and adjusting for BMI, education level, alcohol consumption, fruit and vegetable consumption, smoking status, and pack-years. Odds ratios (OR) and 95% confidence intervals (CI) were reported per one standard deviation increase in log_2_-transformed concentration of each protein. Multiple comparisons were corrected using BH procedure. Significance for the protein–lung cancer association analyses was assessed using nominal *P* < 0.05, with BH-adjusted FDR <0.2 reported in parallel; the two thresholds are reported alongside one another and were not jointly required.

#### Pathway Enrichment Analysis

To explore the biological processes in which air pollution- and lung cancer-associated proteins are involved, we conducted pathway enrichment analysis using the ‘*enrichKEGG*’ function from “*clusterProfiler*” package. To ensure sufficient proteins for input, proteins associated with either air pollution metrics or lung cancer with *P* < 0.05 were included, using a customized background of 484 proteins.

#### Overlapping Proteins and Pathway Identification Using a Meet-In-The-Middle (MITM) Approach

The MITM approach is an exploratory method used to identify potential intermediate biomarkers linking exposure and disease outcomes.^30,31^ This approach has been applied by our group in several high-throughput omics studies investigating various environmental exposures and other health outcomes.^32–34^ The workflow of MITM can be found in the Supporting Information (Figure S2). Because the MITM analysis was designed as an exploratory, hypothesis-generating screen, nominal *P* < 0.05 was used to identify candidate proteins, consistent with previous exploratory MITM analyses.^34–36^ We additionally examined more stringent BH-FDR thresholds of <0.20, <0.10, and <0.05 to assess the robustness of the MITM findings. Because nominal *P* < 0.05 does not control for multiple comparisons, proteins identified only at this threshold were considered hypothesis-generating candidates. Additionally, we identified overlapping biological pathways at nominal *P* < 0.05 that potentially involved in the causal paradigm of air pollution-lung cancer association.

#### High-Dimensional Mediation Analysis

We performed a formal mediation analysis to identify potential protein markers that may mediate the association between individual air pollutant exposure and lung cancer. We used the “*HIMA*” package,^37^ which is dedicated to mediation analysis in a high-dimensional setting where the number of proteins (candidate intermediate biomarkers) is larger than the sample size. The ″*hima*″ function was applied separately for each air pollutant (PM_2_._5_, PM_10_, NO_2_, O_3_, SO_2_, and CO), with all 484 proteins as potential mediators and lung cancer case–control status as the binary outcome. Sure independence screening (SIS) was applied to reduce the input potential mediators from 484 proteins to a subset with the strongest marginal associations with the outcome. By default, the top *d* = [2*n*/*log*(*n*)]­proteins were selected for further analysis, where n represents the sample size (n = 190). Minimax concave penalty (MCP) was used to perform variable selection and coefficient estimation among the screened mediators. All models were adjusted for age, gender, BMI, education level, alcohol consumption, fruit and vegetable consumption, smoking status, and pack-years. Prior to analysis, all air pollutant levels and continuous covariates were standardized to have mean 0 and standard deviation 1. Protein levels had been previously standardized. For HIMA, statistical significance was determined using the package’s Bonferroni-adjusted joint significance criterion of *P* < 0.05. For each significant mediator, HIMA provides the α coefficient representing the association between air pollutant exposure and protein levels adjusting for covariates, β coefficient representing the association between protein levels and lung cancer risk adjusted for air pollutant exposure and covariates, and the indirect effect (αXβ).

As HIMA does not estimate total effects, we performed additional logistic regression analyses to estimate the total effects of six air pollutants (regardless of whether significant mediators were identified) on lung cancer risk, adjusting for covariates. Bootstrap confidence intervals (1000 resamples) were calculated for total effects (all pollutants).

The HIMA package does not accommodate matched case–control designs. Therefore, standard logistic regression rather than conditional logistic regression was used for all exposure-outcome, and mediator-outcome models within the mediation framework.

#### Sensitivity Analyses

To evaluate the robustness of our findings, we performed the following sensitivity analyses. To address concerns of reverse causality, we excluded cases diagnosed within the first year after blood draw. Additionally, we performed a separate analysis by adjusting for cancer status. To address the concerns of residual confounding from smoking, we adjusted for time since quitting smoking.

All analyses were performed using R (Version 4.3.2.).

## Results

### Population Characteristics and Air Pollution Exposure Levels

The 95 matched case–control pairs had a mean age of 70.8 (±5.2) years (Table 1). All participants were White, and 67% were male. Thirty-eight percent of participants maintained a healthy weight (18.5 to <25 kg/m^2^), and 37% attained a bachelor’s degree or higher. Alcohol consumption was reported by 70% of participants. Among controls, 78% were former smokers and 22% were current smokers, with an average smoking history of 28.8 (±22.7) pack years. Among cases, 81% were former smokers and 19% were current smokers, with an average smoking history of 48.8 (±32.3) pack years. Significant differences in pack-years were observed between cases and controls (*P* < 0.05), while other characteristics were comparable between the groups.

**Table 1:** Characteristics of 95 Matched Lung Cancer Case–Control Pairs in the Cancer Prevention Study-II (CPS-II) Nutrition Cohort (N = 190)^a^ ^b^

variable	overall (*N* = 190)	control (*N* = 95)	case (*N* = 95)	*P-value*
age (years)	70.8 ± 5.2	70.8 ± 5.2	70.8 ± 5.2	>0.5
race				
white	190 (100)	95 (100)	95 (100)	NA
sex				>0.5
male	128 (67)	64 (67)	64 (67)	
female	62 (33)	31 (33)	31 (33)	
BMI				>0.5
underweight	2 (1.1)	1 (1.1)	1 (1.1)	
healthy weight	73 (38)	37 (39)	36 (38)	
overweight	92 (48)	46 (48)	46 (48)	
obesity	23 (12)	11 (12)	12 (13)	
education level				0.5
less than high school	15 (7.9)	6 (6.3)	9 (9.5)	
high school graduate	41 (22)	18 (19)	23 (24)	
some college or associate’s degree	63 (33)	31 (33)	32 (34)	
bachelor’s degree and above	71 (37)	40 (42)	31 (33)	
smoking status				>0.5
former	151 (79)	74 (78)	77 (81)	
current	39 (21)	21 (22)	18 (19)	
pack-years	38.8 ± 29.6	28.8 ± 22.7	48.8 ± 32.3	<0.001
alcohol consumption				>0.5
not current drinker	52 (27)	25 (26)	27 (28)	
<1 drink/day	66 (35)	32 (34)	34 (36)	
1 drink/day	36 (19)	22 (23)	14 (15)	
2 or more drinks/day	31 (16)	13 (14)	18 (19)	
unknown	5 (2.6)	3 (3.2)	2 (2.1)	
fruit and vegetable consumption				0.1
first quartile	45 (24)	24 (25)	21 (22)	
second quartile	44 (23)	16 (17)	28 (29)	
third quartile	43 (23)	20 (21)	23 (24)	
fourth quartile	38 (20)	20 (21)	18 (19)	
unknown	20 (11)	15 (16)	5 (5.3)	
year of blood draw				>0.5
1998	6 (3.2)	3 (3.2)	3 (3.2)	
1999	63 (33)	32 (34)	31 (33)	
2000	112 (59)	57 (60)	55 (58)	
2001	9 (4.7)	3 (3.2)	6 (6.3)	
time since last meal				>0.5
less than 2 h ago	100 (53)	48 (51)	52 (55)	
2–4 h ago	78 (41)	42 (44)	36 (38)	
more than 4 h ago	10 (5.3)	4 (4.2)	6 (6.3)	
unknown	2 (1.1)	1 (1.1)	1 (1.1)	

a Continuous variables are reported as mean ± standard deviation, while categorical variables are presented as frequency (%). The detailed selection process, definition, and collection time of covariates can be found in the Supporting Information in the previous air pollution-protein analysis.^22^

b Differences in characteristics between cases and controls were tested using the Wilcoxon rank sum test for continuous variables and Pearson’s Chi-squared or Fisher’s exact test for categorical variables.

The annual mean ± standard deviation concentrations of PM_2.5_, PM_10_, NO_2_, O_3_, SO_2_, and CO in the calendar year of blood draw were 13.0 ± 2.8 μ*g*/*m* ^3^, 21.3 ± 6.1 μ*g*/*m* ^3^, 13.6 ± 6.0 ppb, 48.6 ± 6.8 ppb, 3.6 ± 1.7 ppb, and 0.5 ± 0.2 ppm, respectively (Table S1). All air pollutants levels were similar across cases and controls (Table S1). Pearson correlations among annual average levels of air pollutants ranged from −0.17 to 0.82. Details can be found in Figure S3.

### Multiple Proteins Were Associated with Either Air Pollution Exposure or Lung Cancer Risk

A total of 37, 59, 47, 31, 20, 40, and 15 proteins were associated with PM_2.5_, PM_10_, NO_2_, O_3_, SO_2_, CO, and air pollution mixture, respectively, at *P* < 0.05. Of these, 14 remained significant associations with PM_10_ after multiple testing corrections (FDR < 0.2) (Table S2). Prior studies have comprehensively evaluated the relationships between air pollution and plasma proteins in the CPS-II Nutrition cohort,^22^ as well as between these proteins and lung cancer risk using CPS-II Nutrition and five other cohorts.^26^ When analyzing data from CPS-II Nutrition cohort only, 42 proteins were associated with lung cancer risk (*P* < 0.05), with six remaining significant after multiple testing corrections (FDR < 0.2) (Figure 1). The six proteins were transforming growth factor alpha (TGF-α), WAP four-disulfide core domain (WFDC2), CEA cell adhesion molecule 5 (CEACAM5), interleukin-6 (IL6), C-X-C motif chemokine 17 (CXCL17), and choriogonadotropin subunit beta 3 (CGB3), with elevated plasma levels associated with increased odds of lung cancer. Each of these was identified by the larger, prior study,^26^ except for CGB3 which showed the weakest association among the 6 proteins. Six significant proteins identified above showed positive correlations with each other, except for CEACAM5 and CGB3 (Figure S4). The strongest correlation was observed between TGF-α and CXCL17 (β = 0.67). The model results can be found in the Supporting Information (Excel Tables S1–S8). The weights corresponding to the partial effect of individual air pollutants for proteins in air pollution mixture-protein models (*P* < 0.05) were presented in Excel Table S9.

**Figure 1:**
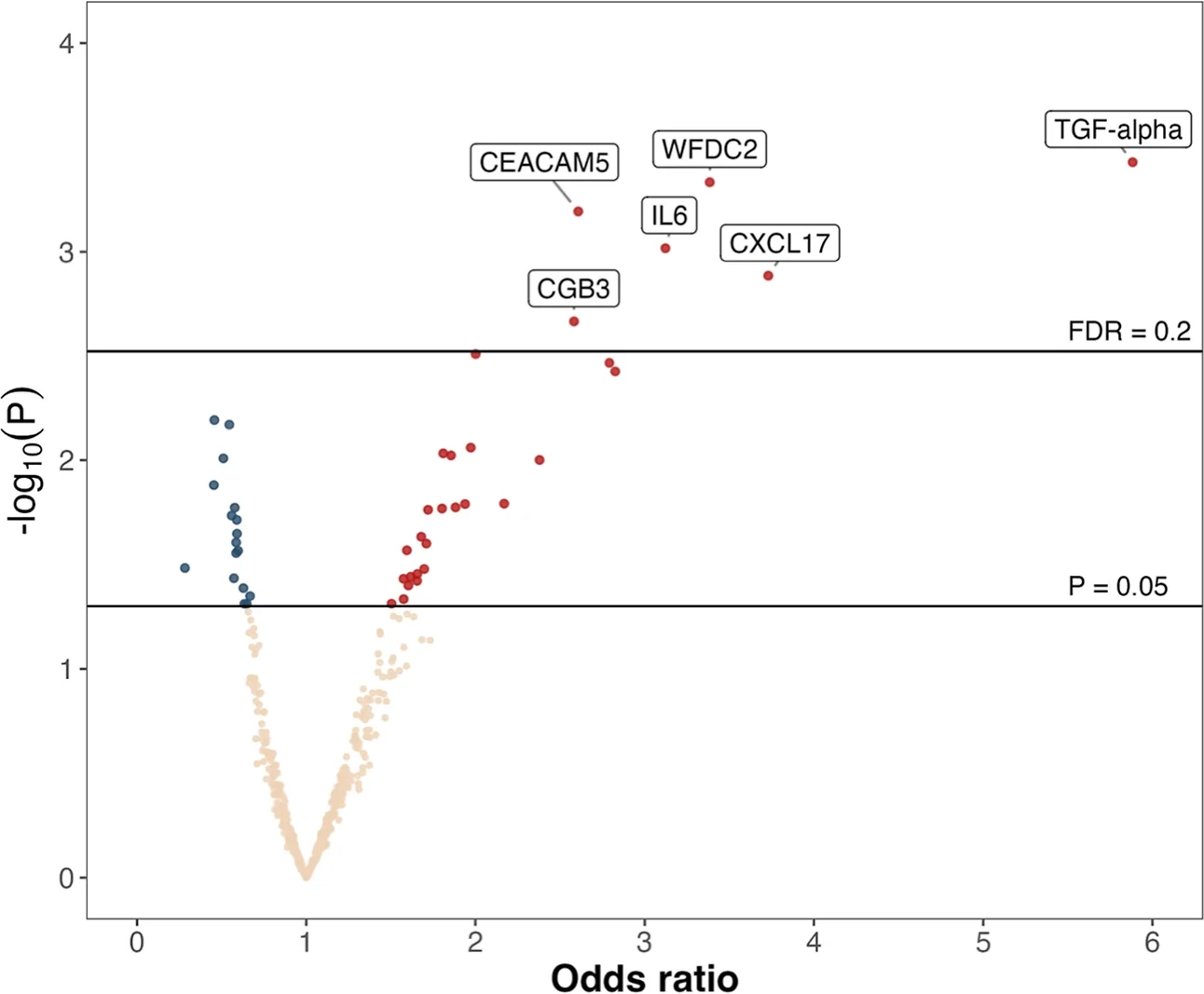
A volcano plot of associations between plasma proteins and lung cancer risk. The *X*-axis represents the odds ratio of lung cancer-protein associations. Odds ratios (95% confidence intervals) per one standard deviation increase in standardized log_2_-transformed concentration of each protein with lung cancer risk were estimated from conditional logistic regression models, matched on age at blood draw, date of blood draw, gender, and race. Models were adjusted for body mass index, education level, alcohol consumption, fruit and vegetable consumption, smoking status, and pack-years. The *Y*-axis represents the negative log_10_ of the unadjusted *P*-value of the lung cancer-protein association. Red dots represent processed protein concentration positively associated with lung cancer risk, while blue dots denote processed protein concentration negatively associated with lung cancer risk.

### Seventeen Proteins Were Associated with Both Air Pollution Exposure and Lung Cancer Risk in MITM

Using the exploratory MITM approach, we identified 17 proteins associated with both air pollution exposure and lung cancer risk (*P* < 0.05) (Figure 2). No overlapping proteins were identified when both associations were required to meet BH-FDR thresholds of <0.20, <0.10, or <0.05. These proteins were therefore considered nominally significant candidate intermediate biomarkers. Since higher air pollution exposure has been thought to increase lung cancer risk, we expected proteins to show concordant associations with both exposure and outcome (i.e., a positive product of the exposure–protein and protein–lung cancer associations). Proteins showing discordant directions, for example, positively associated with air pollution but inversely associated with lung cancer risk, or vice versa, yielding a negative product, were considered less consistent with a straightforward mediation role in the harmful air pollution-lung cancer pathway. Based on this criterion, 11 proteins showed concordant directions (Figure 2A). Specifically, higher air pollution exposures were linked to lower plasma levels of fractalkine (CX3CL1), metalloproteinase domain-containing protein 15 (ADAM15), integrin alpha-V (ITGAV), thiopurine S-methyltransferase (TPMT), fms-related tyrosine kinase 3 ligand (Flt3L), and leptin receptor (LEPR), which were associated with higher lung cancer risk. In contrast, higher air pollution exposures were linked to higher plasma levels of CD300 antigen-like family member E (CD300E), choriogonadotropin subunit beta 3 (CGB3), WFDC2, CXCL17, and tumor necrosis factor receptor superfamily member 6B (TNFRSF6B), which were associated with increased lung cancer risk (Figure 2A). Six additional proteins that show discordant directions with air pollution exposures and lung cancer risk are presented in (Figure 2B).

**Figure 2:**
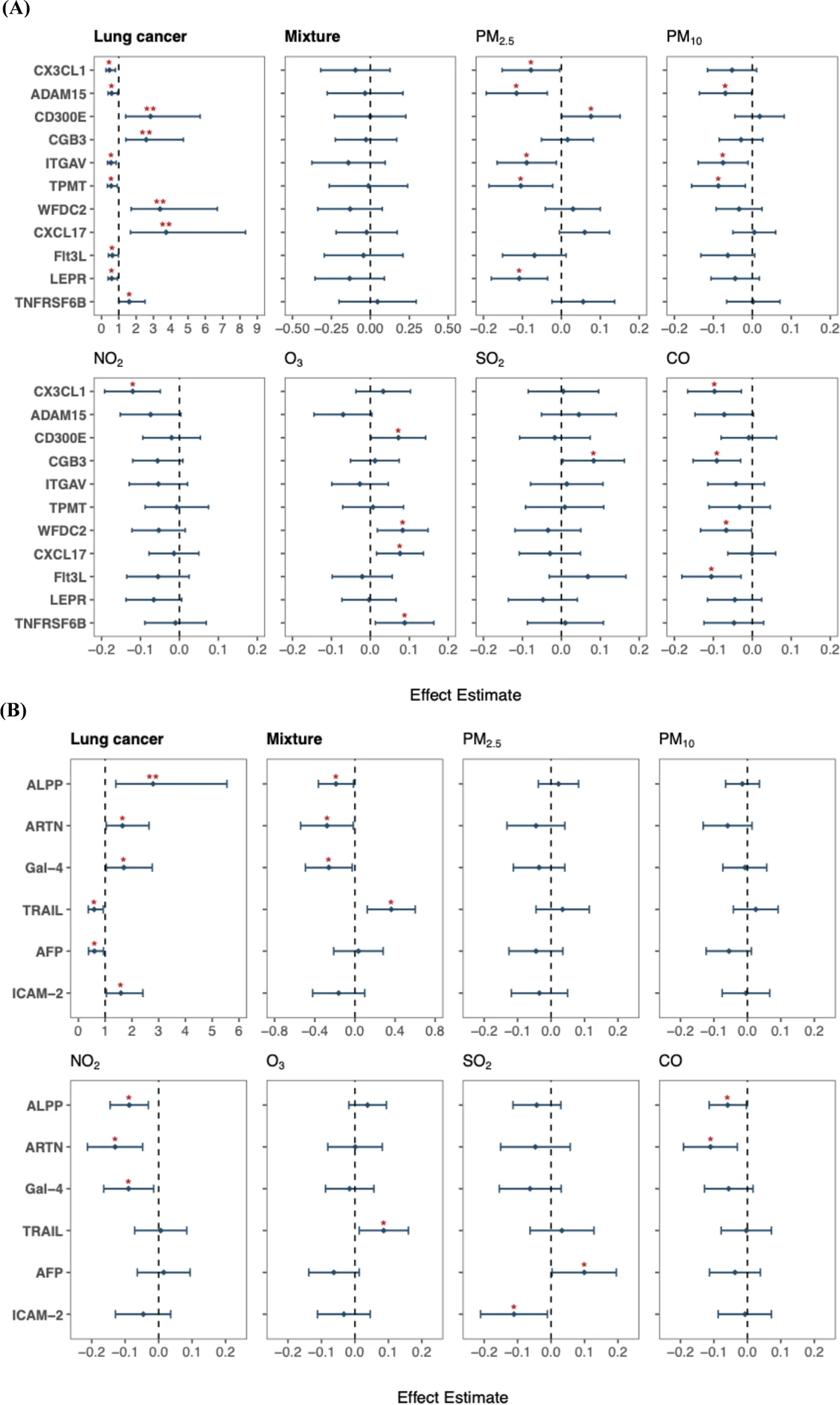
(A) Overlapping proteins showing concordant associations with both air pollution exposure and lung cancer risk at *P* < 0.05 in the Meet-in-the-Middle (MITM) approach. (B) Overlapping proteins showing discordant associations with both air pollution exposure and lung cancer risk at *P* < 0.05 in the MITM approach. Proteins are shown on the *y*-axis. In the lung cancer-protein analysis, the *x*-axis represents odds ratios (OR), with the vertical dashed line at OR = 1. In the air pollution-protein analysis, the *x*-axis represents effect estimates (β), with the vertical dashed line at β = 0. Note: PM_2.5_, fine particulate matter with 2.5 μm or less in diameter; PM_10_, particulate matter with 10 μm or less in diameter; NO_2_, nitrogen dioxide; O_3_, daily 8 h maximum ozone; SO_2_, sulfur dioxide; CO, carbon monoxide; Asterisks: ** FDR <0.2; * unadjusted *P* < 0.05.

### Six Proteins Were Identified as Potential Mediating Proteins in HIMA

Using the HIMA approach, we observed six distinct proteins, CX3CL1, CXCL17, calcium-regulated heat-stable protein 1 (CARHSP1), CGB3, alkaline phosphatase (ALPP), and lysosome-associated membrane glycoprotein 3 (LAMP3), with significant mediation effects (Bonferroni-corrected *P* < 0.05) (Figure 3). The total effects (95% CI) of PM_2.5_, PM_10_, NO_2_, O_3_, SO_2_, and CO on lung cancer risk were 0.42 (0.07, 0.99), 0.37 (−0.01, 0.97), 0.02 (−0.39, 0.42), 0.12 (−0.27, 0.51), −0.02 (−0.51, 0.47), and 0.13 (−0.32, 0.58), respectively, per standard deviation increase in the pollutant levels (Excel Table S10). Among these, only PM_2.5_ showed a statistically significant total effect (*P* < 0.05). The detailed HIMA results of significant proteins were presented in Excel Table S11. Likewise, we expected a mediation (indirect) effect >0, with proteins showing concordant associations with both the exposure and the outcome. Based on this criterion, four proteins met the assumption, which were CX3CL1, CXCL17, CGB3, and CARHSP1 (Figure 3). Notably, three proteins, CX3CL1, CXCL17, and CGB3, were consistently identified by both MITM and HIMA approaches. Importantly, the direction of association among air pollution exposure, protein levels, and lung cancer risk was consistent across two approaches. The PM_2.5_ mediation model failed to converge when pack-years of smoking was included as a covariate. Therefore, no PM_2.5_ mediation results are reported. Although pack-years was scaled, its right-skewed distribution may have contributed to numerical instability in the penalized mediation model. Notably, the model converged successfully after excluding pack-years (results not shown), and the correlation between PM_2.5_ and pack-years was minimal, suggesting that the failure was not due to collinearity but rather to model instability associated with this covariate.

**Figure 3:**
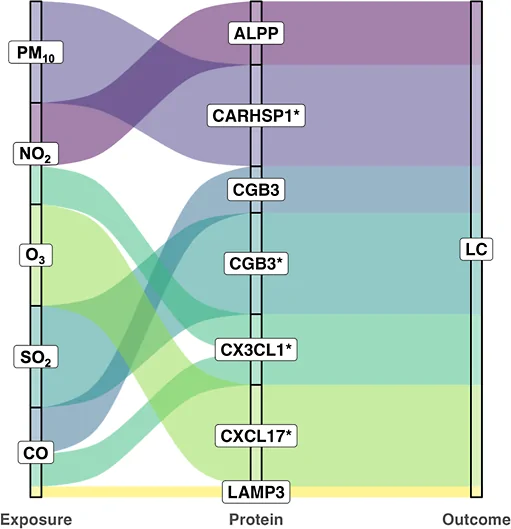
Exposure-specific mediation effects of candidate intermediate proteins on the association between air pollutants and lung cancer risk, as identified in high-dimensional mediation analysis (HIMA). The color stacked bars show the indirect effects of each protein within each pollutant-specific mediation pathway, defined as the indirect effect of each protein divided by the total indirect effect. Different colors represent different proteins. Proteins marked with an asterisk indicate those with a mediation (indirect) effect >0. Mediation effects are interpreted separately for each exposure-outcome relationship and should not be pooled across pollutants. Notably, CGB3 showed a positive indirect effect in the SO_2_ pathway and a negative indirect effect in the CO pathway. Notes: PM_10_, particulate matter with 10 μm or less in diameter; NO_2_, nitrogen dioxide; O_3_, daily 8 h maximum ozone; SO_2_, sulfur dioxide; CO, carbon monoxide; LC, lung cancer.

### Twelve Pathways Were Related to Air Pollution Exposure and Two to Lung Cancer Risk

We included proteins significantly associated with PM_2.5_, PM_10_, NO_2_, O_3_, SO_2_, CO, air pollution mixture, and lung cancer risk at *P* < 0.05, corresponding to 37, 59, 47, 31, 20, 40, 15, and 42 proteins, respectively. Among these, 37, 59, 47, 30, 20, 40, 15, and 42 proteins were successfully mapped to pathway databases. The full measurement panel of 484 proteins was used as the background reference set, of which 483 proteins could be annotated and mapped to the database. The exploratory pathway analysis showed that 12 different pathways were associated with air pollution exposures, while two other pathways were associated with lung cancer risk, which were hematopoietic cell lineage and PI3K-Akt signaling pathway (*P* < 0.05) (Figure 4). In our findings, no significant overlapping pathways were observed associated with both air pollution exposure and lung cancer risk (*P* < 0.05). However, the PI3K-Akt signaling pathway was associated with PM_10_ and lung cancer risk, with a nominal *P* value of 0.05 (Excel Table S12). The detailed pathway results can be found in the Supporting Information (Excel Table S12).

**Figure 4:**
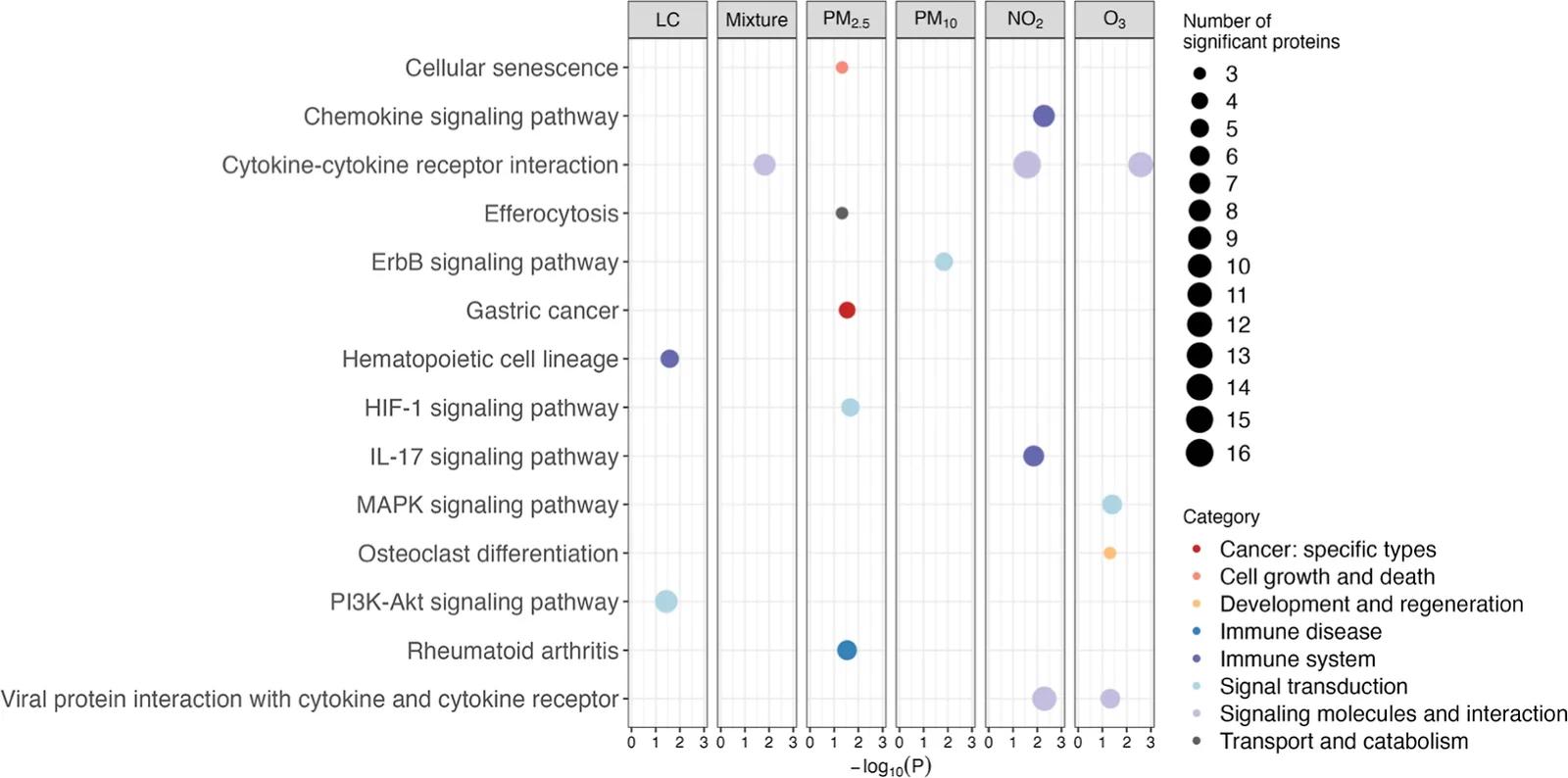
A bubble plot of significant biological pathways associated with either air pollution exposures or lung cancer risk in exploratory pathway analysis (unadjusted *P* < 0.05). Pathways are listed on the *y*-axis, while the -log_10_ of *P* is listed on the *x*-axis. The size of the bubble indicates the number of proteins associated with either air pollution exposures or lung cancer risk at unadjusted *P* < 0.05 within the pathway. The color of the bubbles represents different pathway categories. Abbreviation: LC, lung cancer; Mixture, air pollution mixture; PM_2.5_, fine particulate matter with 2.5 μm or less in diameter; PM_10_, particulate matter with 10 μm or less in diameter; NO_2_, nitrogen dioxide; O_3_, daily 8 h maximum ozone.

### Sensitivity Analyses Results

In the sensitivity analysis of excluding cases diagnosed within the first year after blood draw and their matched controls (n = 71 matched pairs remaining), we observed moderate attenuation in the replication of significant protein associations compared to the main analysis: 54%, 41%, 57%, 71%, 50%, 68%, and 47% of significant proteins for PM_2.5_, PM_10_, NO_2_, O_3_, SO_2_, CO, and the air pollution mixture, respectively, were replicated (*P* < 0.05). For lung cancer risk associations, 62% of significant proteins were replicated. Notably, 41% of proteins showing overlapping associations between air pollution and lung cancer in the main analysis remained significant after excluding cases diagnosed within the first year after blood draw and their matched controls. The substantial proportion of associations that remain significant (41–71% depending on pollutant) suggests that many identified proteins reflect true biological responses to air pollution rather than solely consequences of lung cancer. HIMA did not identify any potential candidate mediators at Bonferroni-corrected *P* < 0.05. Moreover, mediation models for PM_2.5_, NO_2_, and SO_2_ failed to converge. The attenuation and convergence failures observed may reflect reduced statistical power from the smaller sample size, reverse causality, or both; the present analyses cannot distinguish between these explanations. The complete list of significant proteins after excluding cases diagnosed within the first year after blood draw and their matched controls are presented in Excel Table S13.

In the sensitivity analysis of further adjusting for cancer status in the exposure-protein models, the results remained highly consistent with the main analysis. Specifically, we replicated 86%, 90%, 96%, 90%, 95%, 93%, and 87% of the significant protein associations (*P* < 0.05) for PM_2.5_, PM_10_, NO_2_, O_3_, SO_2_, CO, and the mixture, respectively. Effect estimates were largely unchanged between analyses, suggesting that exposure-protein associations are not primarily driven by lung cancer cases. The complete list of significant proteins after adjusting for cancer status are presented in Excel Table S14.

In the sensitivity analysis of further adjusting for time since quitting smoking, we replicated 92%, 83%, 85%, 90%, 70%, 80%, and 80% of the significant protein associations (*P* < 0.05) for PM_2.5_, PM_10_, NO_2_, O_3_, SO_2_, CO, and the mixture, respectively, and 100% for lung cancer risk. Additionally, 94% of proteins showing overlapping associations between air pollution and lung cancer remained significant. HIMA replicated all potential candidate mediators with consistent directions of indirect effects at Bonferroni-corrected *P* < 0.05. However, CGB3 was identified as a potential candidate mediator for CO but not SO_2_ in relation to lung cancer risk. Effect estimates were largely unchanged between analyses. The complete list of significant proteins after adjusting for time since quitting smoking are presented in Excel Table S15.

## Discussion

In the present study, we assessed individual and joint effects of six air pollutants on human proteome and their associations with lung cancer risk among 95 matched lung cancer case–control pairs of ever-smokers from the CPS-II Nutrition Cohort. Using MITM and HIMA, multiple proteins were identified as potential mediating proteins of the association between higher air pollution exposures and increased lung cancer risk. Twelve pathways were associated with air pollution exposure, and two with lung cancer risk. The findings suggest that air pollution exposures may initiate a cascade of biological perturbations that contribute to lung cancer development through mechanisms involving inflammatory chemokine signaling, immune modulation (including evasion and suppression), and altered cell proliferation, migration, and survival (Figure 5).

**Figure 5:**
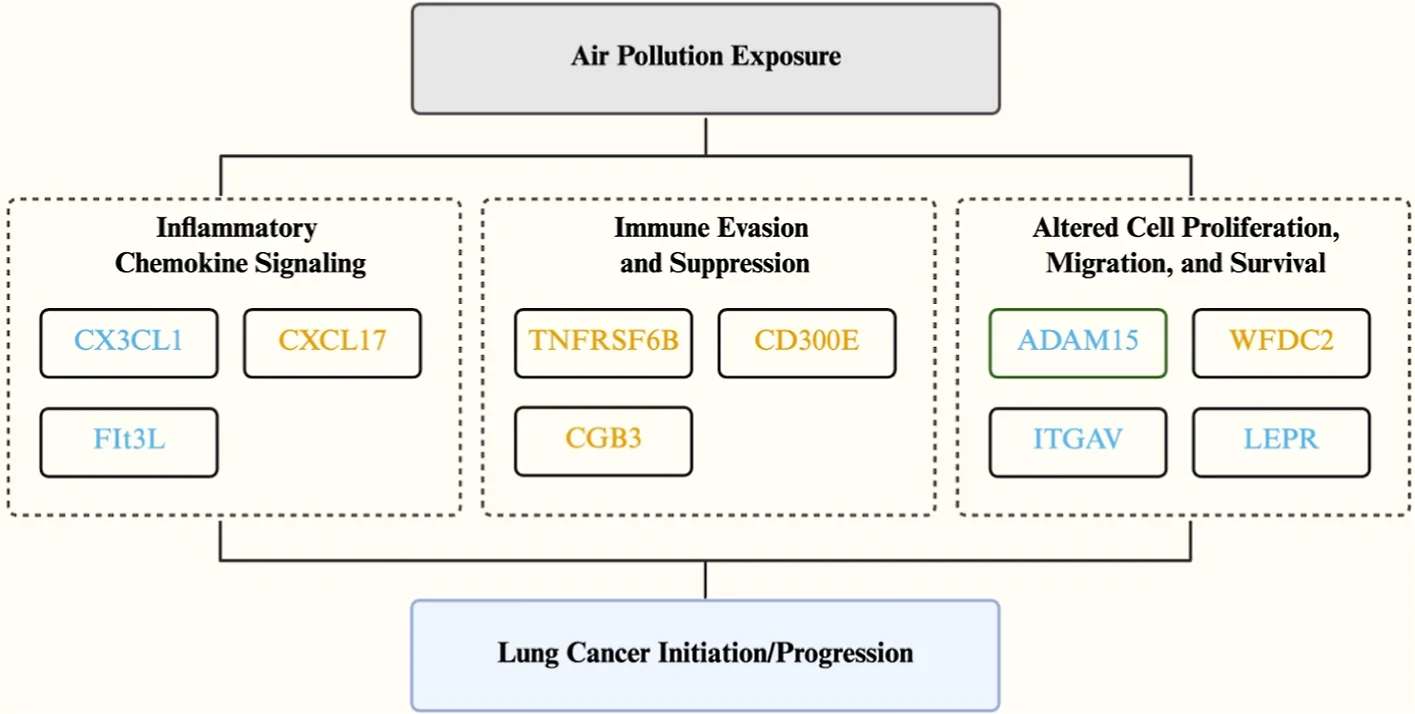
Hypothesized molecular network linking air pollution exposure to lung cancer development. This conceptual diagram integrates proteins identified through Meet-in-the-Middle and high-dimensional mediation analyses. Proteins shown in orange were positively associated with both air pollution exposure and lung cancer risk, whereas proteins shown in blue were negatively associated with both in the present study.

The findings highlight that inflammatory chemokine signaling (e.g., CX3CL1 and CXCL17) may be involved in air pollution-lung cancer association. Decreased plasma levels of CX3CL1 were consistently associated with higher exposure to multiple air pollutants and increased lung cancer risk in the MITM analysis. CX3CL1 can serve as a potent chemoattractant for CX3CR1-expressing cells, including immune-inhibitory myeloid cells and cancer cells, to facilitate their migration.^38,39^ Specifically, the CX3CL1-CX3CR1 axis supports tumorigenesis by driving an influx of CX3CR1^+^ myeloid cells, facilitating migration of multiple CX3CR1^+^ tumor cells including lung cancer, and activating several oncogenic pathways, such as PI3K-Akt and MAPK.^39,40^ The CX3CL1-CX3CR1 axis is being explored as a promising therapeutic target for multiple cancers, including lung cancer, and other diseases.^39,41^ Exploratory pathway analysis results further suggest that the PI3K-Akt signaling pathway may be involved in the association between air pollution and lung cancer.

In addition, we identified several proteins involved in immune checkpoint regulation and immune suppression that may play important roles in the association of air pollution exposure and lung cancer. TNFRSF6B belong to the tumor necrosis factor (TNF) family. TNFRSF6B, a soluble decoy receptor, can bind to apoptosis-inducing ligands, blocking their interaction with respective cell surface receptors and thereby inhibiting apoptosis and promoting cancer cell survival.^42,43^ Additionally, it can modulate the activity of many cell types, including immune cells, potentially fostering immune suppression.^43^ Its overexpression has been found in multiple cancers, including lung cancer,^43^ which is consistent with our findings. Similarly, CD300E is an immunoregulatory receptor primarily expressed on monocytes and macrophages. It has been implicated in facilitating immune evasion and fostering an immunosuppressive tumor microenvironment by triggering pro-tumor T regulatory cells and M2 macrophages.^44^ Its expression varies across tumor types and is notably upregulated in lung cancer, aligning with our findings.^44^ The abnormal expressions of proteins such as CD300E and TNFRSF6B observed in our study, suggest that immune evasion and suppression may contribute to the link between air pollution and lung cancer.

Notably, our findings also highlight several proteins and pathways related to altered cell proliferation, migration, and survival. ADAM15 is a member of the ADAM family that is known for their roles in cell adhesion, migration, signaling, and angiogenesis.^45^ A study found that ADAM15 is overexpressed in nonsmall-cell lung cancer (NSCLC) cell lines and tissues, and its elevated expression is associated with poor prognosis.^46^ ADAM15 promotes NSCLC proliferation and migration via the activation of EGFR and focal adhesion kinase (FAK) signaling pathways.^46^ LEPR is the receptor of leptin (LEP), an obesity-associated adipokine. LEP-LEPR axis is known to regulate appetite and energy metabolism.^47^ Growing evidence indicates that the LEP-LEPR signaling axis contributes to lung cancer progression, including cell proliferation, metastasis, and angiogenesis, by activating several pathways such as PI3K-Akt and MAPK-ERK.^47^ Likewise, ITGAV plays a role in cell proliferation, migration, and immune signaling.^48^ In summary, the abnormal levels of proteins such as ADAM15, LEPR, and ITGAV identified in our study suggest that dysregulation of cell proliferation, migration, and survival may be important for the association between air pollution exposure and lung cancer.

Although the proteins associated with both air pollution and lung cancer are biologically relevant, some of the observed directions of association did not align with the hypothesized biological plausibility. For example, proteins such as CX3CL1 and ADAM15, given their roles in promoting tumorigenesis described above, were expected to show positive associations with both air pollution exposure and lung cancer risk, however we observed inverse associations. Several factors may explain this discrepancy. First, most supporting evidence comes from tumor cell lines and tissue studies, which focus on protein alterations associated with established cancer. In contrast, our study aims to identify circulating protein changes in response to air pollution exposure that occur prior to lung carcinogenesis. Although reverse causation cannot be excluded, making it difficult to distinguish proteins involved in lung cancer development from those altered by occult or established disease, further studies with longer follow-up and repeated biospecimen collection are needed to clarify temporality and strengthen causal inference. Second, cell line models lack the local tumor microenvironment, while tissue studies often capture protein expressions that are influenced by or released from the tumor itself. In our study, the protein profiles were measured in circulating blood, where expression patterns may reflect systemic responses and immune surveillance influenced by various physiological processes, rather than localized tumor-specific activity. Therefore, these circulating levels may not directly reflect concentrations at tumor sites due to their systemic and transient nature. Studies have demonstrated that protein expression profiles differ between blood and tissue samples in patients with breast cancer and renal cell carcinoma.^49,50^ It is worth noting that these studies also highlight that certain proteins are consistently expressed in both sample types, suggesting their potential as blood biomarkers that may reflect tumor biology.^50^ Third, our cross-sectional design captures only a single time point, which may not reflect the temporality between air pollution exposure, changes in protein levels, and lung cancer development. It is possible that certain proteins increase early in the carcinogenic process but decline later due to chronic feedback or immune exhaustion. Future studies should examine longitudinal protein trajectories in response to air pollution to better characterize their temporal dynamics and potential roles in lung cancer etiology.

A recent study characterized the proteomics profiles of lung adenocarcinoma in relation to air pollution among 102 tumor samples from female never-smokers in the Xuanwei area of China.^23^ They found that chronic exposure to coal smoke carcinogens, particularly Benzo­[*a*]­pyrene (BaP), drives tumor-specific activation of the CYP1A1-AhR pathway, with CYP1A1 protein significantly elevated in tumors versus normal tissues. Additionally, tumors harboring BaP-associated EGFR-G719X mutation, exhibited distinct signaling features, including higher expression of MAPK signaling components, specifically MAP2K2 (MEK) and MAPK3 (ERK1). Notably, several key differences exist between our study and theirs. First, the major risk factor identified in their study was exposure to BaP from domestic coal smoke. However, our study focuses on ambient air pollution exposure, examining both individual and combined effects of six air pollutants. Second, comparison of our protein profiles with theirs revealed that only 36% of the proteins measured in our study overlapped with their data set. Notably, CYP1A1, MAP2K2, and MAPK3 were not detected in our study. This limited overlap likely reflects differences in sample types and time of sampling (easily accessible prediagnosis blood-based samples in our study vs invasive tumor samples in theirs), protein profiling platforms, and study populations. As one of the first studies to explore this relationship using prediagnosis blood-based protein measurements, our results warrant validation in longitudinal studies that can establish temporality. Future research should also expand the range of proteins assessed to capture additional relevant molecules.

This study has several strengths. First, this study leverages a well-established prospective cohort with comprehensive data collection. The matched case–control study design helps minimize known confounding effects attributable to matching variables and enhances statistical efficiency by ensuring a balanced distribution of these variables between cases and controls. Second, we employed two complementary approaches, MITM and HIMA, to investigate the potential mediating role of proteins in the association between air pollution exposures and lung cancer risk. Using both methods offers more extensive insights, strengthens robustness, and provides cross-validation of findings. Three proteins, which were CX3CL1, CXCL17, and CGB3, were consistently identified by both MITM and HIMA approaches. Third, this study accounts for the joint effects of multiple air pollutants on plasma protein levels in MITM but not in HIMA. However, no overlapping proteins were observed between air pollution mixtures and lung cancer risk in MITM, likely due to limited statistical power from the small sample size and uncertainties arising from simultaneously considering multiple pollutants derived from models with various performances.

This study has several limitations. First, the cross-sectional design limits the ability to establish a causal relationship between air pollution exposure and plasma protein levels, which is an inherent assumption of mediation analysis. Moreover, although the total effect was not statistically significant for most pollutants except PM_2.5_ in this sample, which likely reflects the limited size of this nested case–control subset given the significant associations reported in the full CPS-II cohort,^51^ mediation analysis may still identify biologically plausible intermediate proteins that reflect specific exposure-related pathways and warrant further investigation. Additionally, all lung cancer cases were diagnosed within three years of blood draw, which may not fully capture the relationship between chronic exposure and lung cancer risks. However, after excluding the cases diagnosed within 1 year after blood draw, the substantial proportion of associations that remain significant suggests that many identified proteins reflect true biological responses to air pollution rather than solely consequences of lung cancer. Second, annual pollutant concentrations in the calendar year of blood collection were used as proxies for longer-term relative exposure, supported by high year-to-year correlations. However, this metric may include postcollection exposure and does not establish strict temporal ordering. Longer-term or lagged exposures could not be evaluated because of limited historical pollution data and residential history. Exposure assignment based on a single address and calendar year may also have introduced largely nondifferential misclassification, likely attenuating associations under conventional assumptions. Moreover, our analysis used total PM as a surrogate for PM exposure without accounting for specific sources or chemical components that are important determinants of PM toxicity. Third, we did not consider potential effect modification by smoking history, which may influence the biological responses to air pollution. Additionally, we lacked data on occupational exposures and indoor air pollution exposures, which may confound the associations of interests. Although we adjusted for smoking status and pack-years in the main analysis, as well as time since quitting smoking in sensitivity analysis, residual confounding by smoking behaviors cannot be entirely ruled out. Fourth, several proteins identified by MITM and HIMA showed discordant associations with air pollution exposure and lung cancer risk, which were considered less consistent with a straightforward mediation role in the harmful air pollution-lung cancer pathway. Such discordance may arise from compensatory biological responses that counteract air pollution-induced harmful effects, reverse causation due to early subclinical tumor, residual confounding by shared risk factors, or timing of protein measurement relative to exposure and disease onset. Fifth, long-term storage of blood samples may affect protein stability. A study using the same Olink platform found that storage at −80 °C for up to 26 years affected approximately 15% of proteins (18/122), explaining 4.8–34.9% of variance in those affected proteins.^52^ Given our lower storage temperature and comparable duration, we expect a better preservation of proteins in our study. Any protein degradation would likely introduce nondifferential measurement error, potentially attenuating observed associations toward the null. Sixth, we simply assumed additive model with linear exposure effects for interpretability and did not consider nonlinear or interactive pollutant relationships. Seventh, because the HIMA package does not accommodate matched case–control designs, we used standard rather than conditional logistic regression in the mediation analysis. Consequently, the analysis did not fully account for the matched study design, which may have reduced statistical efficiency and affected the precision of the estimated associations. In main and sensitivity analyses, some HIMA mediation models failed to converge. The convergence failures observed may reflect reduced statistical power, reverse causality, or both, and these explanations cannot be distinguished with the present data. Lastly, the findings may not be generalizable to never-smokers, younger populations, racially or ethnically diverse populations, or populations exposed to different pollutant mixtures. Future studies should include larger and more diverse populations, longer follow-up, and repeated biospecimen collection; evaluate effect modification by smoking; incorporate pollutant mixtures into mediation analyses using approaches such as HIMA2 or multiple-exposure mediation;^53,54^ examine nonlinear and interactive effects among pollutants; and assess the reproducibility and robustness of the identified protein biomarkers. Accordingly, the present findings should be interpreted cautiously and considered hypothesis-generating.

Taken together, this study offers new insights into the mechanisms by which air pollution exposure may alter the human proteome and potentially contribute to lung cancer development. It identifies specific proteins and pathways related to air pollution-lung cancer association that warrant further investigation and independent validation.

## Supplementary Material





## Notes

Table S1 for the annual average exposure levels of six air pollutants of the study population. Table S2 for the number of proteins associated with either air pollution exposures or lung cancer at different thresholds. Figure S1 for study population exclusion criteria and final sample size. Figure S2 for workflow of the meet-in-the-middle (MITM) approach. Figure S3 for Pearson correlations among annual average levels of six air pollutants. Figure S4 Pearson correlations among six significant proteins associated with lung cancer risk (FDR < 0.2) (PDF)
Excel Table S1 for associations between annual average fine particulate matter (PM_2.5_) exposure level and each protein. Excel Table S2 for associations between annual average particulate matter with 10 μm or less in diameter (PM_10_) exposure level and each protein. Excel Table S3 for associations between annual average nitrogen dioxide (NO_2_) exposure level and each protein. Excel Table S4 for associations between annual average daily 8 h maximum ozone (O_3_) exposure level and each protein. Excel Table S5 for associations between annual average sulfur dioxide (SO_2_) exposure level and each protein. Excel Table S6 for associations between annual average carbon monoxide (CO) exposure level and each protein. Excel Table S7 for associations between annual average air pollution mixture exposure and each protein. Excel Table S8 for associations between lung cancer risk and each protein. Excel Table S9 for weights corresponding to the partial effect of individual air pollutants for significant proteins in air pollution mixture-protein models (*P* < 0.05). Excel Table S10 for total effects of each air pollutant on lung cancer risk with bootstrapped 95% confidence intervals. Excel Table S11 for the detailed high dimensional mediation analysis results. Excel Table S12 for biological pathways associated with either air pollution exposure or lung cancer risk (unadjusted *P* < 0.05). Excel Table S13 for proteins associated with either air pollution or lung cancer risk (*P* < 0.05) in sensitivity analysis of removing cases diagnosed within the first year after blood draw and their matched controls. Excel Table S14 for proteins associated with either air pollution (*P* < 0.05) in sensitivity analysis of further adjusting for cancer status. Excel Table S15 for proteins associated with either air pollution or lung cancer risk (*P* < 0.05) in sensitivity analysis of further adjusting for years since quitting smoking (XLSX)

## Notes

Disclaimer: Where authors are identified as personnel of the American Cancer Society and International Agency for Research on Cancer/World Health Organization, the authors alone are responsible for the views expressed in this article and they do not necessarily represent the decisions, policy or views of the American Cancer Society or the American Cancer Society–Cancer Action Network or International Agency for Research on Cancer/World Health Organization.
