# Supplementary material for: Analysis of the Potential Mediating Role of Plasma Proteins in the Association Between Air Pollution Exposure and Lung Cancer Risk Among Ever-Smokers
Source: Environ Sci Technol. 2026 Oct 19;60(43):30401–13. doi: 10.1021/acs.est.6c07407 (PMC13738795; doi:10.1021/acs.est.6c07407)
Supplement: Supplementary file 1 [file es6c07407_si_001.pdf]

Supplementary Materials

Analysis of the Potential Mediating Role of Plasma Proteins in the Association between Air Pollution Exposure and Lung Cancer Risk among Ever-smokers

Ziyin Tang[1], Donghai Liang[1,*], Jeremy A. Sarnat[1], W. Ryan Diver[2,3,4], Todd M. Everson[1], Emily Deubler[2], Youran Tan[1], Aparna H. Kesarwala[6], Michelle C. Turner[3,4,5], Mattias Johansson[7], Hilary A. Robbins[7], Carmen J. Marsit[1], Alpa V. Patel[2], Ying Wang[2,*]

[1]Gangarosa Department of Environmental Health, Rollins School of Public Health, Emory University, Atlanta, GA, USA, 30322

[2]Department of Population Science, American Cancer Society, Atlanta, GA, USA, 30303

[3]Barcelona Institute for Global Health (ISGlobal), Barcelona, Spain, 08003

[4]Universitat Pompeu Fabra (UPF), Barcelona, Spain, 08003

[5]CIBER Epidemiología y Salud Pública (CIBERESP), Madrid, Spain, 28029

[6]Department of Radiation Oncology, Emory University School of Medicine, Atlanta, GA, USA, 30322

[7]Genomic Epidemiology Branch, International Agency for Research on Cancer, Lyon, France, 69366

***Corresponding authors:**

Donghai Liang, PhD

Associate Professor, Gangarosa Department of Environmental Health

Rollins School of Public Health, Emory University

1518 Clifton Rd NE

Atlanta, GA, 30322

donghai.liang@emory.edu

Ying Wang, PhD

Senior Principal Scientist, Department of Population Science

American Cancer Society, Inc.

270 Peachtree Street NW Suite 1300

Atlanta, GA 30303

ying.wang@cancer.org

Competing Interests

The authors declare that they have no competing interests.

Number of pages	8
Number of figures	4
Number of tables	2

Table S1. Annual average exposure levels of six air pollutants of the study population.

**Air pollution exposure levels** [a]	**Overall** (N = 190)	**Controls** (N = 95)	**Cases** (N = 95)	***P*-value** [b]
$PM_{2.5}$ ($\mu g/m^3$)	13.0 ± 2.8	12.6 ± 2.9	13.3 ± 2.6	0.090
PM_{10} ($\mu g/m^3$)	21.3 ± 6.1	20.4 ± 5.8	22.1 ± 6.3	0.089
NO_2 (ppb)	13.6 ± 6.0	13.8 ± 5.7	13.5 ± 6.4	0.3
O_3 (ppb)	48.6 ± 6.8	48.4 ± 7.2	48.8 ± 6.3	0.4
SO_2 (ppb)	3.6 ± 1.7	3.6 ± 1.7	3.6 ± 1.7	0.9
CO (ppm)	0.5 ± 0.2	0.5 ± 0.2	0.5 ± 0.2	0.6

[a] Annual average air pollution exposure levels in the calendar year of blood draw. $PM_{2.5}$, fine particulate matter with 2.5 micrometers or less in diameter; PM_{10}, particulate matter with 10 micrometers or less in diameter; NO_2, nitrogen dioxide; O_3, daily 8h maximum ozone; SO_2, sulfur dioxide; CO, carbon monoxide.

[b] The air pollution exposure levels of controls and cases were compared using the Wilcoxon rank sum test.

Table S2.The number of proteins associated with either air pollution exposures or lung cancer at different thresholds. The total number of proteins included in the analyses was 484.

**Exposures/Outcome**	**Threshold**	
	unadjusted *P* < 0.05	BH-corrected FDR < 0.2
$PM_{2.5}$	37	0
PM_{10}	59	14
O_3	47	0
NO_2	31	0
SO_2	20	0
CO	40	0
Mixture	15	0
Lung cancer	42	6

Note: $PM_{2.5}$, fine particulate matter with 2.5 micrometers or less in diameter; PM_{10}, particulate matter with 10 micrometers or less in diameter; NO_2, nitrogen dioxide; O_3, daily 8h maximum ozone; SO_2, sulfur dioxide; CO, carbon monoxide; Mixture, air pollution mixture; BH, Benjamini-Hochberg procedure; FDR, false discovery rate.

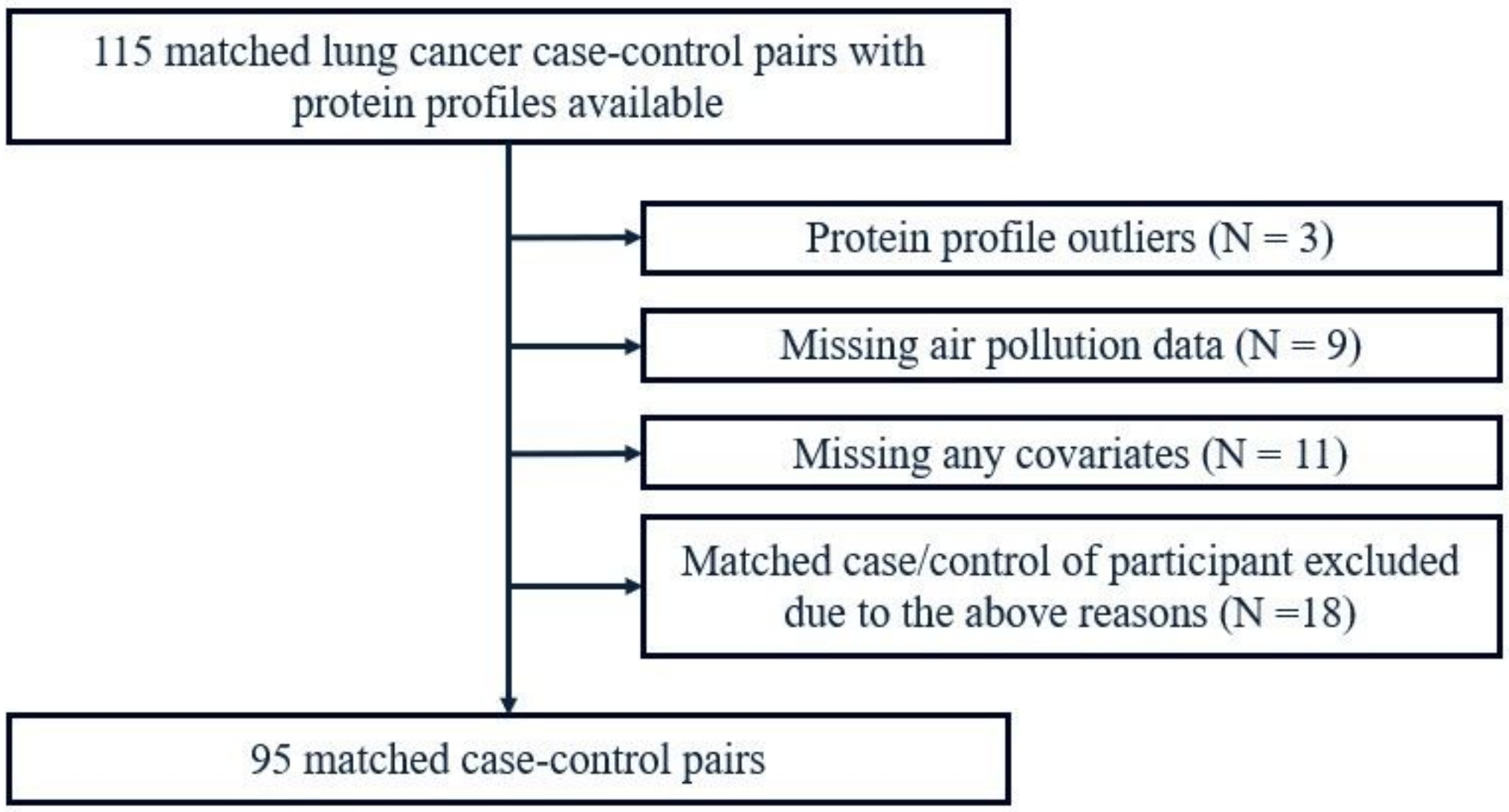

Figure S1. Study population exclusion criteria and final sample size.

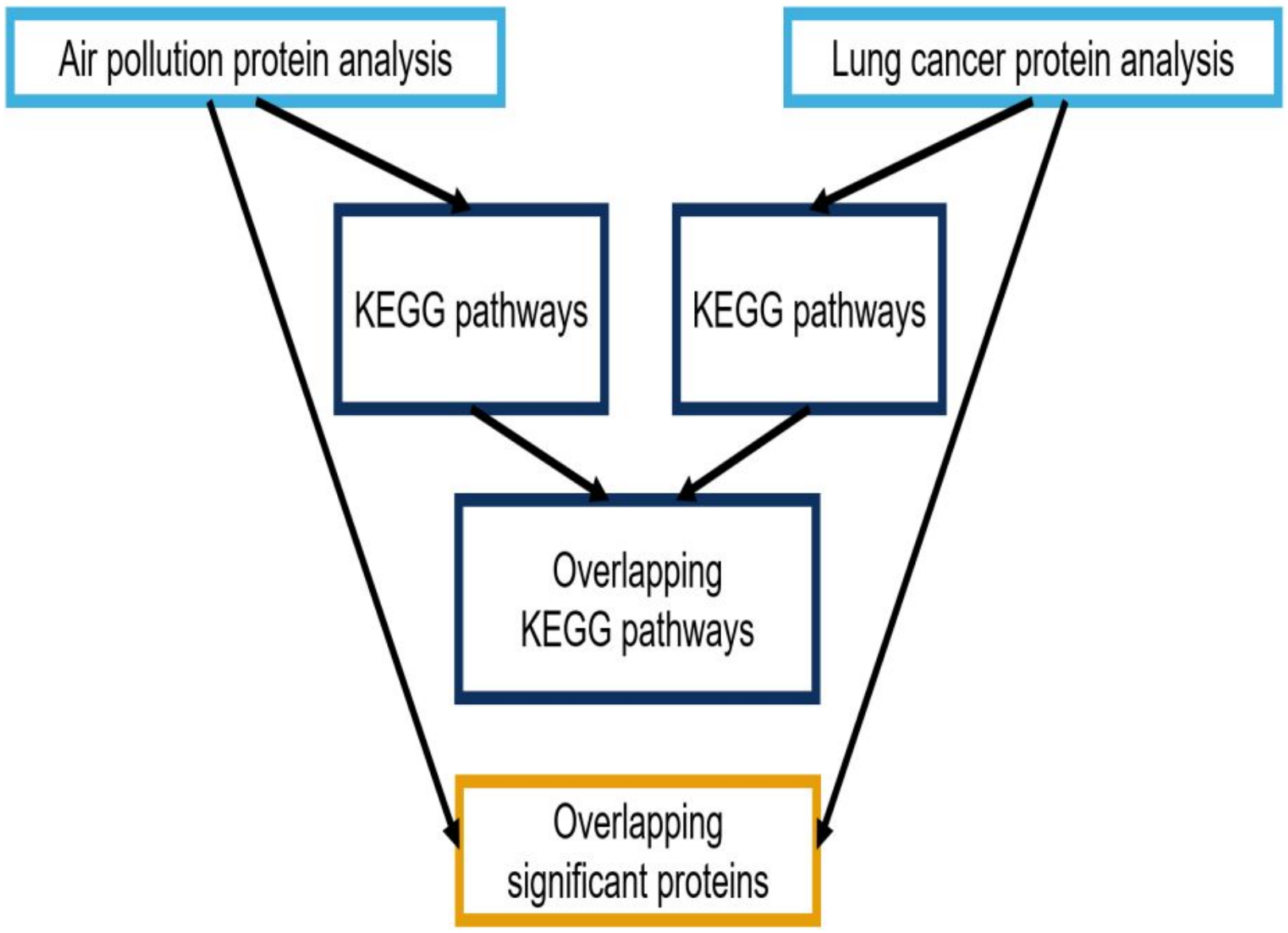

Figure S2. Workflow of the meet-in-the-middle (MITM) approach.

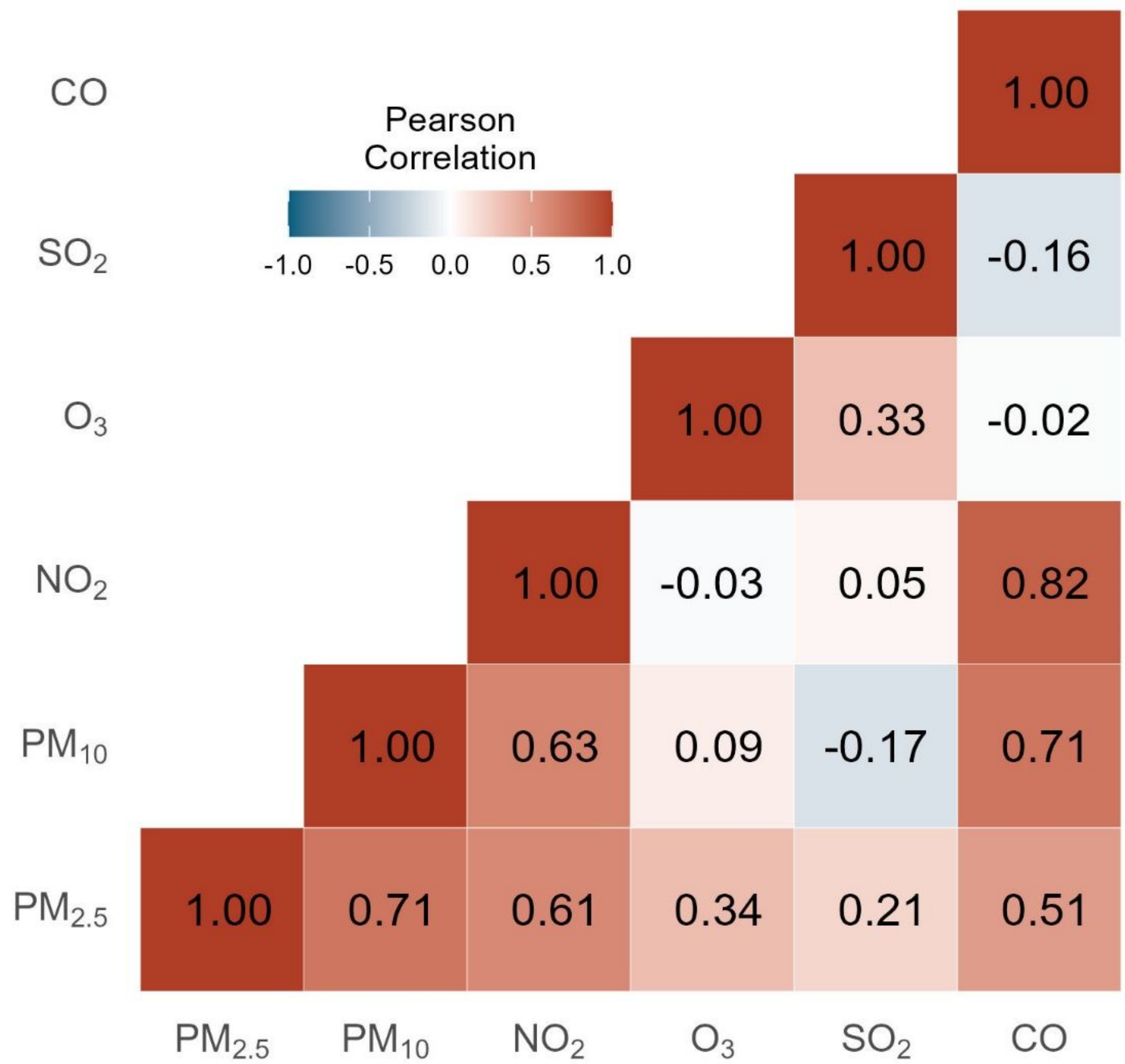

Figure S3. Pearson correlations among annual average levels of six air pollutants.

Note: $PM_{2.5}$, fine particulate matter with 2.5 micrometers or less in diameter; PM_{10}, particulate matter with 10 micrometers or less in diameter; NO_2, nitrogen dioxide; O_3, daily 8h maximum ozone; SO_2, sulfur dioxide; CO, carbon monoxide.

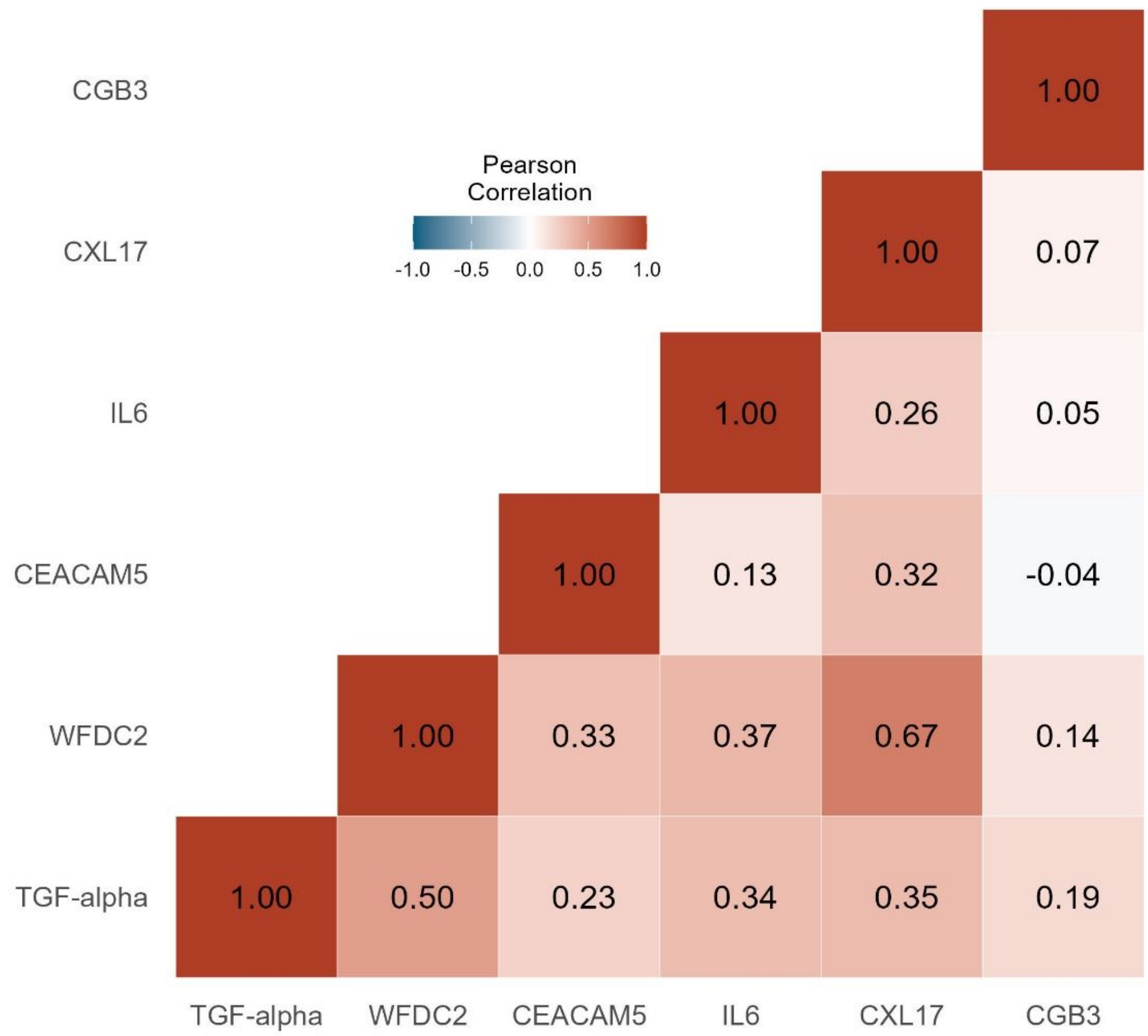

Figure S4. Pearson correlations among six significant proteins associated with lung cancer risk (false discovery rate (FDR) < 0.2).
